# Knowledge creation elements for enhancing community resilience towards disaster: A Delphi study

**DOI:** 10.4102/jamba.v13i1.1137

**Published:** 2021-10-25

**Authors:** Rina S. Oktari, Khairul Munadi, Rinaldi Idroes, Hizir Sofyan, Bokiraiya Latuamury

**Affiliations:** 1 Graduate School of Mathematics and Applied Science, Universitas Syiah Kuala, Banda Aceh, Indonesia; 2 Tsunami and Disaster Mitigation Research Centre (TDMRC), Banda Aceh, Indonesia; 3 Department of Family Medicine, Faculty of Medicine, Universitas Syiah Kuala, Banda Aceh, Indonesia; 4 Department of Electrical and Computer Engineering, Faculty of Engineering, Universitas Syiah Kuala, Banda Aceh, Indonesia; 5 Department of Chemistry, Faculty of Mathematics and Natural Sciences, Universitas Syiah Kuala, Banda Aceh, Indonesia; 6 Department of Statistics, Faculty of Mathematics and Natural Sciences, Universitas Syiah Kuala, Banda Aceh, Indonesia; 7 Department of Forestry, Faculty of Agriculture, University of Pattimura, Ambon, Indonesia

**Keywords:** disaster, community resilience, knowledge management, spiral socialisation-externalisation-combination- internalisation (SECI), consensus

## Abstract

Knowledge capacity plays a vital role in building community resilience to disasters. However, the problem is that there is no resilience framework that integrates the knowledge creation process. This article introduces a new framework for increasing community resilience based on knowledge creation theory (KCT). This research aims to define the elements that support the Knowledge Creation for Community Resilience (KCCR) and to gain consensus from experts on these factors. This study was conducted using semi-structured interviews with five panellists and three rounds of Delphi technique to determine the assessment of 26 factors (including six additional factors) that have been identified by experts (30, 18 and 11 experts in rounds I, II and III, sequentially). The data analysis was carried out in several stages, and included Spearman’s Rank Correlation Coefficient, consensus appraisal and interrater agreement (IRA) statistical evaluation. The result of the agreement level (AL) analysis shows that the majority of the constructs (96.15%) are in the ‘moderate strong’ category. This study shows that there is a significant consensus (with IRA index [*a*_*wg*(1)_] ranging from 0.529 to 1), and panellists confirm the significance of all the key constructs. Consensus was gained from experts on seven elements that support the KCCR. This study establishes a systematic, operational and multidimensional KCCR framework that combines the concepts of knowledge creation, community resilience and disaster preparedness. This framework can be used as a qualitative instrument or guidance to build community resilience based on knowledge creation and a quantitative tool for measuring community resilience in facing disasters.

## Introduction

The frequency with which worldwide disasters are occurring has increased in the last few decades. According to the Emergency Events Database (EM-DAT), in 2019 at least 396 disasters were reported, whereby this number is slightly above the average number of disasters that occurred in the last 10 years (343 disasters). Catastrophic events killed at least 11 755 people, affected another 95 million and caused US $ 130 billion in losses. Floods and storms were the biggest contributor (68%) of the total number of people who were affected by disaster. At the regional level, Asia is the largest continent in terms of the number of disasters (40%), the total deaths (45%), and the proportion of the population affected by disasters (74%) globally (EM-DAT 2020).

The increasing occurrence and magnitude of damage triggered by natural hazards require strategies at the local, regional and global scales to increase community resilience. The Hyogo Framework for Action (HFA) 2005–2015 and the Sendai Framework for Disaster Risk Reduction (SFDRR) 2015–2030 are global frameworks for disaster risk reduction (DRR). Both frameworks emphasise the importance of DRR efforts through capacity building in knowledge, education and preparedness (UNISDR 2005, 2015).

Several studies have also shown that knowledge capacity plays a vital function in building community resilience to disasters, especially in making the right decisions for individuals and groups or organisations (Fujieda & Kobayashi 2013; Oktari et al. 2015, 2018). Local and indigenous knowledge plays an important role in saving lives during a disaster, thus transmitting it (knowledge, values and skills) intergenerationally is imperative (Oktari et al. 2015). Weichselgartner and Pigeon (2015) also emphasised the important role of knowledge in DRR, which then recommends further studies to analyse current and newly disaster knowledge creation and transform knowledge into wisdom. However, there is no current resilience framework that integrates the knowledge creation process. Knowledge creation is the essence of knowledge-based management (KM) (Nonaka & Takeuchi 1995). Originally, knowledge creation theory (KCT) (Nonaka 1994) was designed to facilitate the management system of the organisation or enterprise. However, as a key model for knowledge management, its use has now been expanded in the public domain, particularly in addressing social problems and community empowerment (Nonaka & Nishihara 2018). Applying KM in disaster management can have significant implications, especially in improving performance in disaster management (Oktari et al. 2020). Thus, it is crucial to develop a knowledge-based-community resilience framework as a basis for decision-making and to ensure the transgenerational transmission of disaster knowledge (Oktari et al. 2021).

This research aims to develop a new community resilience framework based on the knowledge creation concept and identify the elements that support the framework. The Delphi process was deployed to collect opinions on the proposed framework and gain consensus from experts who have experience in the area of disaster, climate change, community development, and development of communication. The specific objectives of this research are to: (1) identify elements of knowledge creation based on expert judgement, (2) identify elements of community resilience based on expert assessments, (3) analyse the importance level (IL) of each element of knowledge creation and community resilience that has been identified, (4) analyse the agreement level (AL) of each element of knowledge creation and community resilience that has been identified and (5) develop the new knowledge creation for the community resilience model based on the results of the Delphi study conducted.

### Identification of knowledge creation factors to enhance community resilience

#### Knowledge creation theory

Knowledge creation theory was introduced in the 1980s from several case studies of manufacturing companies in Japan (Nonaka 1994). Research using KCT was implemented in multinational companies and small- and medium-sized enterprises (SMEs), both inside and outside Japan, including those companies belonging to the government, non-governmental organisations and society. The KCT was then developed substantively by synthesising several theories and concepts in an interdisciplinary manner, including philosophy, psychology, cognitive science and neuroscience (Mihalca et al. 2008).

Knowledge creation is defined as a dynamic and collaborative process that is built based on the experiences of individuals and organisations, resulting in a fundamental and ongoing conversion of tacit knowledge into explicit knowledge, and vice versa to crystallise knowledge (Nonaka & Takeuchi 1995). Explicit knowledge and tacit knowledge complement reciprocally, where both have an important role in the knowledge creation process.

According to Nonaka’s knowledge creation theory, there are three (3) components involved in the knowledge creation process, namely: (1) socialisation-externalisation-combination- internalisation (SECI) Spiral Process, (2) common context *Ba* for the knowledge creation process and (3) knowledge assets. Those three components should have natural and dynamic interaction with each other (Nonaka, Toyama & Konno 2000).

The SECI spiral process consists of four (4) modes of knowledge conversion, namely (1) socialisation; involves converting tacit knowledge into additional tacit knowledge through experience, observation, imitation and practice, (2) externalisation; comprises converting tacit knowledge into explicit knowledge in the form of expression as in language (written or verbal) and other symbolic manifestations (such as hieroglyphs, videos, picture albums, dancing, theatre production, etc.), (3) combination; consists of converting explicit knowledge into additional explicit knowledge, using media such as documents, meetings, telephone conversations and computer communication (4) internalisation; means converting explicit knowledge into tacit knowledge and is firmly grounded in traditional learning and understanding notions (Nonaka et al. 2000). This SECI process is different from several other notions of the knowledge management processes, for example, (1) the production-codification-distribute-utilisation process (Ford 2004); (2) the process of creating, acquiring, transferring and applying knowledge (Alavi & Leidner 2001) and (3) dynamic growth of competency improvement (Zollo & Winter 2002).

The word *Ba* comes from Japanese, which means a place, space or domain. The concept of *Ba* was introduced by Nonaka et al. (2000) to explain that the knowledge creation process must have a unique context based on time, space and relationships with other individuals. *Ba* provides the energy, quality and place to convert individual knowledge to SECI’s spiral process. *Ba* is an existential place where individuals share their respective contexts and create new meanings through interaction.

Knowledge assets are input, output and moderating factors based on the knowledge creation process (Nonaka et al. 2000). There are four (4) types of knowledge assets: (1) experiential knowledge assets, consisting of shared knowledge built through direct experience amongst members of the organisation and between members of the organisation and other individuals outside the organisation, (2) conceptual knowledge assets, consisting of explicit knowledge articulated through images, symbols and language, (3) systemic knowledge assets, consisting of explicit knowledge that is systematised and packaged and (4) routine knowledge assets, composed of common tacit knowledge embedded in everyday actions and organisational practices (Nonaka et al. 2000).

The knowledge assets are assembled and shared in *Ba*, where an individual’s tacit knowledge is converted and augmented by the SECI spiral of knowledge. Additionally, the three components of the knowledge creation process must be strengthened under clear leadership to enable continuous and dynamic creation of knowledge (Nonaka et al. 2000; Yoo et al. 2020).

#### Knowledge creation in the context of community resilience

The KCT (Nonaka 1994) aims to answer questions related to how organisations effectively create new knowledge. According to the theory of Nonaka (1994), interactions that occur continuously between individuals who exchange knowledge actively and explicitly would be a meaningful force to encourage the growth of new ideas and concepts in organisations.

The concept of a ‘knowledge ladder’ (North & Kumta 2014) provides a measured assessment of information to be converted to ongoing knowledge and competency. This concept explains that each stage of the ‘knowledge ladder’ is built on (1) a bottom-up approach that represents the operational mechanisms of information and knowledge and (2) a top-down approach that reflects the strategic steps to define the capabilities of an organisation and its affiliates, so that it can increase the spirit of competition. In the disaster circumstances, the success of the main knowledge ladder is in resolving how to directly link knowledge management and resilience, so that individuals can make the right decisions for saving lives in the face of disasters and adopt adaptive behaviour in coping with the consequences of climate change.

**FIGURE 1:**
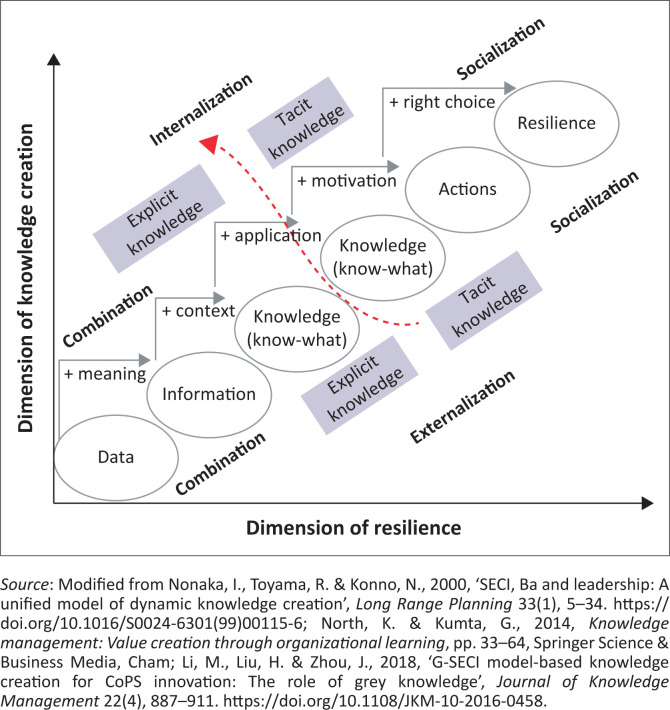
A two-dimensional model of knowledge creation to increase resilience.

In the present study, Nonaka’s knowledge creation model (Nonaka 1994), after elaboration with the North and Kumta (2014) knowledge ladder concept, can better explain how the role of knowledge as an intangible advantage will enhance sustainability, performance and creativity in increasing disaster resilience, as illustrated in Figure 1.

This two-dimensional model consists of several stages on the knowledge ladder. The transformation of data into useful information is the first rung of the ladder. The second rung of the ladder is the transformation of data into knowledge (know-what). The process of transitioning from knowledge (know-what) to knowledge (know-how) is the third rung of the ladder. In this case, the internalisation process to convert explicit knowledge into tacit knowledge is needed to apply the knowledge possessed. The fourth rung emphasises how knowledge (know-how) would motivate to take action. The last rung explains how the knowledge owned by an individual could be the basis for individuals to make the right choice to achieve resilience. Each rung is essentially a set of explicit and tacit knowledge. The formation of explicit knowledge sets comes from continuous repeated learning. At the same time, the results of each rung are also part of the knowledge set on the next rung. Simultaneously, individual tacit knowledge is generated through reoccurred learning during the process.

Knowledge creation is attached to the individual context itself (including social interaction), as well as spatial, cultural and historical contexts (Nonaka et al. 2000). Some of these contexts are the basis for translating various information into a concrete and meaningful message or tool. Individuals initiate knowledge creation processes that are then distributed to the organisation (Finley & Sathe 2013). As explained in the SECI model, knowledge creation theory explains the dialectical process that is driven by tacit and explicit knowledge, which involves a spiral model and a continuous process, as well as emphasises the importance of knowledge sharing (Hislop 2013; Nonaka & Von Krogh 2009).

In the perspective of building community resilience towards disasters, the family unit is the smallest organisation and is an integral unit in maintaining resilience. Therefore, the application of KCT developed (Nonaka 1994) can also be applied at the family level, thus treating the family as a micro-sized organisation.

#### Community resilience

When viewed as an entire context, resilience can be appropriately regarded as a system attribute. A ‘stable’ system was previously regarded as robust, solid and reluctant to change (Manyena 2006; McEntire et al. 2002). A stable system is presently defined as adaptable to stress and can maintain its properties under various settings (Tiernan et al. 2019).

Previous research has identified several components of resilience, including religious affiliation, place of attachment, spirituality, ethnicity, culture, social beliefs, community education, community empowerment, practices, social networks, familiarity with local services, physical and economic security, economic development, social capital, information and communication and community competence (Bastaminia, Rezaei & Saraei 2017; Cutter, Burton & Emrich 2010; Cutter et al. 2008; Norris et al. 2008; Oktari et al. 2020, 2021; Sherrieb, Norris & Galea 2010).

Community resilience is defined as the capacity, hopes and beliefs of the community with which to withstand, overcome and control major challenges that occur, such as socio-economic changes or catastrophic events (whether caused by natural or human-made hazards), by increasing resources, competence and connectedness. The resources in question are biological, psychological, social and spiritual resources that can be accessed and utilised to overcome the direct impacts and consequences of trauma, so that they can support long-term recovery and healing, and can be preserved from generation to generation (Landau 2010).

The family acts as an integral unit of change. Therefore, the intervention that is carried out in the community also refers to individuals, families and social organisations within that community, which includes history, culture, economy and the physical environment. Hence, in this case, family resilience has a close relationship with community resilience (Landau 2010).

Resilience is a multifaceted, multidimensional, multilevel dynamic process. It is also a reciprocal interlinkage of individual, family, sociocultural and institutional stimuli during a lifetime and across generations. Continuous crises and stressful events will affect the whole family unit and all of its members individually, and will pose a risk, not only for individual instability but also for relational tension and family disintegration (Walsh 2015).

Family processes can help families overcome the influence of unfavourable circumstances on all individual members, the interactions between them and the survival of the family component. Dynamic family processes can promote resilience by resisting stress, establishing strengths and mobilising resources to accelerate affirmative adaptation. Meanwhile, a maladaptive response can increase the susceptibility and likelihood of individual discord, relational disorders and family destruction. All individuals and families have the ability to increase their resilience. Efforts should be made by maximising that ability by promoting their best efforts, improving core processes and leveraging resources (Walsh 2015).

**FIGURE 2:**
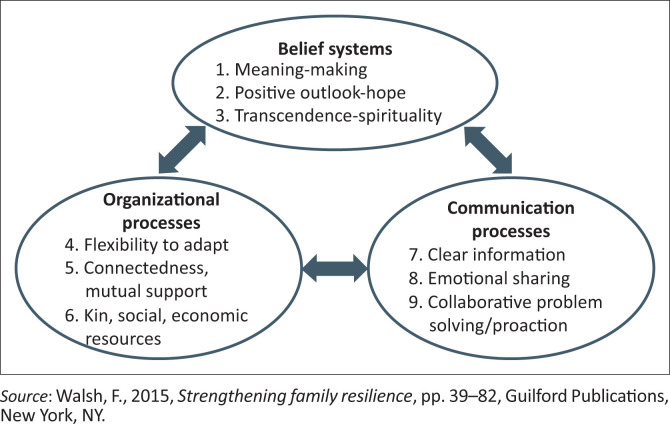
Family resilience framework.

The family resilience framework (see Figure 2) is a conceptual map for (1) identifying and targeting the main family mechanisms that minimise stress and vulnerability in high-risk circumstances, including disasters; (2) promoting long-term recovery and also immediate recovery from crisis conditions and (3) empowering families to cope with prolonged adversity. This framework outlines nine key processes in family resilience and organises the processes conceptually in three domains of family function, namely: (1) family belief system, (2) organisational process and (3) problem-solving communication process. The nine key processes are interactive and synergistic, within and across domains (Walsh 2015).

This framework is neither a typology nor a set of fixed features of the resilient family. Instead, it describes a dynamic process that involves strengths and resources that families can access and use to increase family resilience. Some processes may be more (or less) relevant and useful in many adverse situations and various social and cultural contexts. Family members can map various pathways in resilience depending on the values, sources, challenges and goals of each member (Walsh 2015).

When families encounter severe life crises and struggles, the notion of family resilience reinforces the capacity that exists in the family for stability, improvement and advancement. Family resilience creates a foundation for a strength-based approach to practise. Because families have a range of opportunities, obstacles and coping mechanisms, there are several paths to family resilience. Therefore, in building family resilience in the face of pressure or risk, an understanding of the main processes in mobilising existing resources is needed (Walsh 2015).

## Materials and methods

**FIGURE 3:**
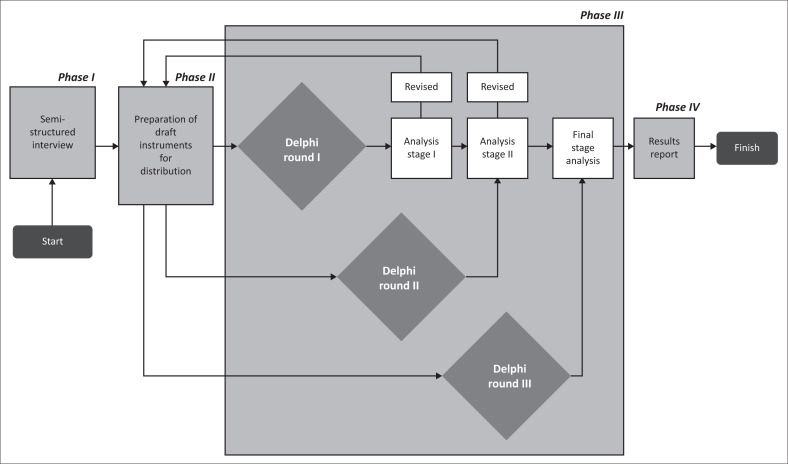
Flowchart of the research method of this study.

This research was carried out in several steps (see Figure 3) to achieve the study objectives. (1) In the first stage, semi-structured interviews were carried out with five knowledge management and community resilience experts (both from academic and government) who validated the factors that had been previously identified. This process is also carried out to explore additional factors, reduce redundancy and eliminate overlapping factors. (2) In the second stage, developing an initial research questionnaire was carried out to determine normality, reliability and building consensus using the Delphi technique. (3) In the third stage, the Delphi technique was used, and consists of three rounds to gather the necessary information and reach consensus. (4) In the fourth stage, there is a discussion of the Delphi analysis results and the conclusions of the research.

This study uses a Delphi technique, which consists of three rounds. The first round aims to (1) assess the factors that influence knowledge creation in increasing community resilience and (2) identify the relationship between these factors. The second round aims to (1) reassess the factors that influence knowledge creation in increasing community resilience and (2) assess the new factors that experts added in the first round and (3) assess the relationship between these factors. The third round aims to (1) determine new factors added by the experts in the second round and (2) identify their relationship.

Inclusion criteria were established to select experts to be involved in the Delphi study, including a willingness to take part in the research; have experience in disaster management or climate change or community empowerment or sustainable development; have at least the level of Bachelor’s Degree as an educational background. The Delphi process is carried out until consensus is reached or for a maximum of three rounds, considering that the experts might be tired in responding or because of their busy schedule (Keeney, Hasson & McKenna 2006). Experts who were not willing to take part in round II were not invited to engage in round III. To complete the Delphi process, the expert must provide feedback in all three rounds.

A total of 30 experts from Indonesia, Japan and Germany who matched the inclusion criteria participated in the first round of this Delphi study, 18 experts continued to attend the second round and only 11 experts participated in the third round (see Table 1). The experts in the Delphi study include academics, governments and practitioners in the field of disasters and climate change. The majority of panellists have expertise in the area of DRR (90%) and more than 10 years of experience (63.34%).

**TABLE 1:** Characteristics of the experts in the Delphi study.

Variable	Round I (*N* = 30)		Round II (*N* = 18)		Round III (*N* = 11)	
	*n*	%	*n*	%	*n*	%
**Gender**						
Male	21	70.00	13	72.22	8	72.73
Female	9	30.00	5	27.78	3	27.27
**Category**						
Government	8	26.67	4	22.22	2	18.18
Practitioner	15	50.00	10	55.56	5	45.45
Academic	7	23.33	4	22.22	4	36.36
**Education**						
Bachelor	6	20.00	4	22.22	1	9.09
Master	15	50.00	9	50.00	5	45.45
Doctoral	9	30.00	5	27.78	5	45.45
**Expertise**						
Disaster risk reduction	27	90.00	16	88.89	11	100.00
Climate change adaptation	10	33.33	7	38.89	5	45.45
Community development	10	33.33	9	50.00	5	45.45
Development communication	9	30.00	6	33.33	3	27.27
**Experience (year)**						
Less than 5	3	10.00	2	11.11	1	9.09
Between 5 and 10	7	23.33	4	22.22	2	18.18
More than 10	19	63.33	11	61.11	7	63.64

Experts were invited to assess the factors that had previously been identified through the literature review using an online survey. After the first Delphi round, the six new factors that were suggested by the panellists were included, together with the factors that were obtained from the literature review results.

Consensus in the Delphi study is usually determined by the percentage of agreement with a particular response, followed by the percentage of participants who rated the item at the upper extreme of the Likert scale (Diamond et al. 2014; Foth et al. 2016). Thus, the assessment in this Delphi study uses a Likert scale with five category points as follows: 1 = not important; 2 = slightly important; 3 = moderately important; 4 = important and 5 = very important. The analysis in this Delphi study includes Spearman’s Rank Correlation Coefficient, consensus analysis (Mode, Mean, Median) and analysis of IL and AL.

Data about changes in opinions or judgements of experts in this study were analysed using the *Spearman’s Rank Correlation Coefficient* (*r_s_*), by applying the following formula (Kalaian & Kasim 2012):

$$r_{s}=1-\frac{6\sum D_{i}^{2}}{n\left(n^{2}-1\right)}$$ [Eqn 1]

where *D_i_*= the difference between the answer ratings on item *i* in the two Delphi rounds; and *n* = number of experts.

The *r_s_*value is between the ranges of −1 and +1, where a value of +1 indicates a perfect positive relationship between ratings on two consecutive rounds. The closer the *r_s_*value to +1, the more significant the relationship between ratings on two successive rounds. The closer the *r_s_*value is to 0, the smaller the correlation, which indicates no correlation between ratings on two consecutive rounds. The closer the *r_s_* value to −1, the more significant the relationship between the assessments in the opposite direction, indicating that expert agreement was not reached on two consecutive rounds.

The overall consensus of the experts was assessed using the Mode value (MdV), Mode score (MdS), Mean (M), Median (Mdn), and the number of percentage ratings ≥ 4 for each construct in rounds 2 and 3. A positive consensus is obtained if the percentage of item scores assessed is in the ‘important’ and ‘very important’ categories (scores 4 and 5) with a proportion of ≥ 70% (Bisson et al. 2010; Suzuki et al. 2012).

Although when giving an assessment the experts cannot assume the same interval between these values, the intensity of the ratings given in two successive rounds can be said to be comparable to the ratings amongst other consecutive categories using a Likert scale (Cohen, Manion & Morrison 2013). This shows that the level interval used to interpret the degree of importance for each construct can be re-assigned to fit the mean score between two sequential assessments (Zahoor et al. 2017).

The IL is calculated based on the average value with the following categories: 0 < 1.5 = ‘not important’, 1.51–2.5 = ‘slightly important’, 2.51–3.5 = ‘moderately important’, 3.51–4.5 = ‘important’ and > 4.5 = ‘very important’ (Gunduz & Elsherbeny 2020).

In the final round, the intensity of agreement is checked by interrater agreement (IRA) analysis and a threshold of significance is established for each construct. The IRA is an alternative approach for assessing the magnitude of agreement in the Delphi study. This process eliminates bias because of the influence of scale, sample size and a number of experts. The IRA index (*a_wg_*_(1)_) between experts is calculated using the following equation (Brown & Hauenstein 2005).

$$a_{wg(1)}=1-\frac{2\times SD^{2}}{\left[(H+L)\times M-M^{2}-H\times L\right]\times k/(k-1)}$$ [Eqn 2]

where *SD* = standard deviation for each factor; *H* = highest value (scale); *L* = lowest (scale) value; *M* = average expert rating for one factor and *k* = number of experts in each round.

If the value of *a_wg_*_(1)_ is equal to 1, it indicates a perfect agreement. The IRA calculation results are interpreted with the AL as follows: 0.00–0.30 = ‘very weak’, 0.31–0.50 = ‘weak’, 0.51–0.70 = ‘moderately strong’, 0.71–0.90 = ‘strong’ and 0.91–1.00 = ‘very strong’ (Brown & Hauenstein 2005).

## Results

### Factors influencing knowledge creation to increase resilience

Based on the description in the earlier section, in the first round of Delphi, experts evaluated five elements and 20 factors contribute to increasing the community’s resilience based on knowledge creation. (1) The first element, the knowledge creation process or SECI process, consists of socialisation (Kcp1), externalisation (Kcp2), combination (Kcp3) and internalisation (Kcp4). (2) The second element, the *Ba* shared context, which consists of originating *Ba* (Ba1), dialoguing/interacting *Ba* (Ba2), systemising/cyber *Ba* (Ba3) and exercising *Ba* (Ba4). (3) The third element, knowledge assets, which consists of experiential knowledge assets (Ka1), conceptual knowledge assets (Ka2), systemic knowledge assets (Ka3) and routine knowledge assets (Ka4). (4) The fourth element, the enabler of knowledge creation, which consists of intention (Kce1), autonomy (Kce2), fluctuations and creative chaos (Kce3), redundancy (Kce4) and requisite variety (Kce5). (5) The fifth element, community resilience, consists of belief system (Cr1), organisational processes (Cr2) and communication processes (Cr3).

The sixth and the seventh elements, along with six factors, were added in the second round of Delphi, based on the semi-structured interview results (in the first round). (6) The sixth element, factors affecting knowledge, consists of internal factors (If) and external factors (Ef). (7) The seventh element, disaster preparedness, which consists of knowledge (Dp1), emergency planning (Dp2), warning system (Dp3) and resource mobilisation (Dp4).

Detailed descriptions of seven elements and 26 factors that influence knowledge creation in increasing community resilience are presented in Table 2.

**TABLE 2:** Identified constructs of knowledge creation and resilience.

Elements	Construct	Description	Code	Reference
Knowledge creation process (SECI process)	Socialisation	Conversion of tacit knowledge into additional tacit knowledge of disaster (e.g. experience, observation, imitation and practice)	Kcp1	(Kruke & Olsen 2012; Martín-de-Castro, López-Sáez & Navas-López 2008; Nonaka et al. 2000)
	Externalisation	Conversion of tacit knowledge into explicit knowledge of disaster	Kcp2	
	Combination	Conversion of explicit knowledge into additional explicit knowledge of disaster	Kcp3	
	Internalisation	Conversion of explicit knowledge into tacit knowledge of disaster and ensure it is firmly grounded in traditional notions of learning and understanding	Kcp4	
Shared context of *Ba (Ba is a Japanese term to explain a virtual, physical and mental context in knowledge creation)*	Originating *Ba*	Share direct experiences and build tacit knowledge of disaster in a communal environment	Ba1	(Nonaka et al. 2000; Nonaka & Konno 1998; Schmitt 2016; Von Krogh & Geilinger 2014)
	Dialoguing/interacting *Ba*	Express tacit knowledge of disaster through dialogue and reflection	Ba2	
	Systemizing/Cyber *Ba*	Organise a relevant concept of explicit knowledge of disaster into a model, prototype or narrative	Ba3	
	Exercising *Ba*	Practising the model so that it becomes tacit knowledge of disaster	Ba4	
Knowledge assets	Experiential	Shared tacit knowledge of disaster that is built through a direct experience collectively	Ka1	(Năftănăilă 2012; Nonaka et al. 2000; Nonaka, Umemoto & Senoo 1996)
	Conceptual	Explicit knowledge of disaster expressed through pictures, icons and language	Ka2	
	Systemic	Formalised and bundled explicit knowledge of disaster, for example, explicitly stated technology, product specifications, manuals, databases and legally protected intellectual property (such as patents and licenses)	Ka3	
	Routine	Tacit knowledge of disaster which is habituated and embedded in daily actions and practices	Ka4	
Knowledge creation enablers	Intention	Direction and aspirations that drive the process of disaster knowledge creation (attitudes, subjective norms and control of perceived behaviour)	Kce1	(Lloria & Peris-Ortiz 2014; Nonaka et al. 1996; Roth 2003; Von Krogh & Geilinger 2014)
	Autonomy	Think rationally and reach a decision	Kce2	
	Fluctuation and creative chaos	Sense of crisis and more creative in facing external threat (expressed in the way of thinking, mental models, paradigms of values)	Kce3	
	Redundancy	A way of transferring tacit knowledge of disaster that allows one to see a variety of ways to articulate information	Kce4	
	Requisite variety	Facilitates individual access to a variety of disaster-related information quickly, flexibly and can be combined in different ways	Kce5	
Factors affecting knowledge	Internal factors	Individual internal factors (age, intelligence)	If	(Ni et al. 2015; Rahman 2016)
	External factors	Individual external factors (education, environment, social culture, information, experience, motivation)	Ef	(Espesor 2018; Nakamura, Umeki & Kato 2017; Rahman 2016; Seneviratne, Baldry & Pathirage 2010)
Community resilience	Belief system	The ability to find meaning in difficult conditions, maintain an optimistic outlook and have a strong spiritual conviction to deal with disasters	Cr1	(Landau 2010; Ricciardelli 2018; Walsh 2015)
	Organisational patterns	Flexibility, connectedness and having access to the necessary social and economic resources in a disaster situation	Cr2	
	Communication processes	The clarity in communicating, expressing emotions openly and collaborative problem-solving skills towards disaster	Cr3	
Disaster preparedness	Knowledge	Understanding disaster risk	Dp1	(Atta-ur-rahman 2015; Oktari et al. 2020)
	Emergency plan	Plan for evacuation, rescue and aid	Dp2	
	Early warning	Access to warning signs and distribution of information of disaster warning	Dp3	
	Resource mobilisation	The ability to mobilise available resources, both human resources (HR), as well as funding and essential infrastructure for emergencies	Dp4	

SECI, Socialisation-Externalisation-Combination-Internalisation.

### Analysis of the expert’s judgment change

Data about changes in opinions or judgements of experts in this study were analysed using the *Spearman’s Rank Correlation Coefficient* (*r_s_*). The *r_s_* value is between the ranges of −1 and +1, where a value of +1 indicates a perfect positive relationship between ratings on two consecutive rounds. The closer the *r_s_* value to +1, the more significant the relationship between ratings on two successive rounds. The closer the *r_s_* value is to 0, the smaller the correlation, which indicates no correlation between ratings on two consecutive rounds. The closer the *r_s_* value to −1, the more significant the relationship between the assessments in the opposite direction, indicating that expert agreement was not reached on two consecutive rounds.

Based on the table for the critical value of *Spearman’s Rank Correlation Coefficient*, at *a* = 0.05 and *n* = 11, the value is 0.536. Analysis results shown in Table 3 show that all *r_s_* scores (ranging from 0.958 to 1) are higher than critical values. Thus, the relationship is considered to be significantly strong.

**TABLE 3:** Results of the *Spearman’s rank correlation coefficient* from two Delphi rounds.

No.	Construct	$\sum D_{i}^{2}$	*r_s_*	No.	Construct	$\sum D_{i}^{2}$	*r_s_*
1	Kcp1	2	0.988	14	Kce2	3	0.982
2	Kcp2	3	0.982	15	Kce3	3	0.982
3	Kcp3	3	0.982	16	Kce4	4	0.976
4	Kcp4	7	0.958	17	Kce5	3	0.982
5	Ba1	4	0.98	18	If	4	0.98
6	Ba2	5	0.97	19	Ef	2	0.99
7	Ba3	4	0.98	20	Dp1	2	0.987
8	Ba4	4	0.98	21	Dp2	2	0.987
9	Ka1	1	0.99	22	Dp3	2	0.987
10	Ka2	1	0.99	23	Dp4	2	0.987
11	Ka3	1	0.99	24	Cr1	4	1
12	Ka4	1	0.99	25	Cr2	5	1
13	Kce1	3	0.982	26	Cr3	4	1

*D_i_* the difference between the answer ratings on item *i* in the two Delphi rounds; *r_s_*, Spearman’s Rank Correlation Coefficient.

### Consensus analysis

The overall consensus of the experts was assessed using the Mode value (MdV), Mode score (MdS), Mean (M), Median (Mdn) and the number of percentage ratings ≥ 4 for each construct in rounds two and three.

A positive consensus is obtained if the percentage of item scores assessed is in the ‘important’ and ‘very important’ categories (scores 4 and 5) with a proportion of ≥ 70%. Of the total 33 constructs assessed, 26 (78.78%) constructs reached positive consensus in round I.

As shown in Table 4, it can be seen that in round II, as many as 14 constructs (53.84%) reached a consensus of 100% of experts, 11 constructs (42.30%) reached consensus from 90.90% of experts and one construct (3.84%) reached consensus from 72.72% of experts. In round III, all 26 constructs (100%) achieved a consensus with the proportion of 100% experts. From these results, it can be inferred that the consensus analysis in this study produces positive results with a proportion of 100% for the overall constructs.

**TABLE 4:** Results of consensus analysis of the Delphi study.

Construct	Round II					Round III				
	MdV	MdS	M	Mdn	≥ 4 (%)	MdV	MdS	M	Mdn	≥ 4 (%)
Kcp1	5	63.6	4.64	5	100.00	5	81.8	4.82	5	100
Kcp2	5	63.6	4.64	5	100.00	5	90.9	4.91	5	100
Kcp3	4	54.5	4.45	4	100.00	5	72.7	4.73	5	100
Kcp4	5	45.5	4.27	4	90.90	5	72.7	4.73	5	100
Ba1	5	54.5	4.45	5	90.90	5	81.8	4.82	5	100
Ba2	5	54.5	4.45	5	90.90	5	90.9	4.91	5	100
Ba3	5	54.5	4.55	5	100.00	5	90.9	4.91	5	100
Ba4	5	63.6	4.64	5	100.00	5	100.0	5.00	5	100
Ka1	5	72.7	4.73	5	100.00	5	81.8	4.82	5	100
Ka2	5	72.7	4.73	5	100.00	5	81.8	4.82	5	100
Ka3	5	72.7	4.73	5	100.00	5	81.8	4.82	5	100
Ka4	5	72.7	4.73	5	100.00	5	81.8	4.82	5	100
Kce1	4	54.5	4.27	4	90.90	5	54.5	4.55	5	100
Kce2	4	54.5	4.27	4	90.90	5	54.5	4.55	5	100
Kce3	4	54.5	4.27	4	90.90	5	54.5	4.55	5	100
Kce4	4	54.5	4.27	4	90.90	5	63.6	4.64	5	100
Kce5	4	54.5	4.27	4	90.90	5	54.5	4.55	5	100
If	5	54.5	4.27	5	72.72	5	63.6	4.64	5	100
Ef	5	72.7	4.64	5	90.90	5	81.8	4.82	5	100
Dp1	5	54.5	4.55	5	100.00	5	72.7	4.73	5	100
Dp2	5	63.6	4.64	5	100.00	5	81.8	4.82	5	100
Dp3	5	54.5	4.55	5	100.00	5	72.7	4.73	5	100
Dp4	5	54.5	4.55	5	100.00	5	72.7	4.73	5	100
Cr1	4	54.5	4.45	4	100.00	5	81.8	4.82	5	100
Cr2	4	72.7	4.09	4	90.90	5	54.5	4.55	5	100
Cr3	4	54.5	4.27	4	90.90	5	63.6	4.64	5	100

MdV, Value Mode; MdS, Mode Score (%); M, Mean; Mdn, Median.

### Analysis of importance level and agreement level

In the final round, the intensity of agreement is checked by IRA analysis and a threshold of significance is established for each construct. The IRA is an alternative approach for assessing the magnitude of agreement in the Delphi study. This process eliminates bias because of the influence of scale, sample size and a number of experts. If the value of IRA index (*a_wg_*_(1)_) between experts is equal to 1, it indicates perfect agreement.

Table 5 shows ratings and IL, as well as IRA analysis for all factors from the third round of Delphi Study. The average expert assessment of the constructs ranges from 4.55 to 5.0. Thus, the analysis of the IL indicates that all constructs are in the category of ‘very important’. The result of the AL analysis shows that the majority of the constructs (96.15%) are in the ‘moderately strong’ agreement category. There is one construct that is categorised as a ‘very strong’ agreement or reaching a ‘perfect agreement’, namely Ba4. The percentage agreement after this third round reaches 100%, hence the results of the IRA analysis support the consensus results that were reached in the previous analysis, and confirm that all 26 key factors make a significant contribution to knowledge creation in increasing resilience.

**TABLE 5:** Results of importance level analysis and interrater agreement in the third round.

Construct	M	IL	SD	*α*_*wg* (1)_	AL
Kcp1	4.82	Very important	0.405	0.566	Moderately strong
Kcp2	4.91	Very important	0.302	0.529	Moderately strong
Kcp3	4.73	Very important	0.467	0.606	Moderately strong
Kcp4	4.73	Very important	0.467	0.606	Moderately strong
Ba1	4.82	Very important	0.405	0.566	Moderately strong
Ba2	4.91	Very important	0.302	0.529	Moderately strong
Ba3	4.91	Very important	0.302	0.529	Moderately strong
Ba4	5	Very important	0	1	Strong
Ka1	4.82	Very important	0.405	0.566	Moderately strong
Ka2	4.82	Very important	0.405	0.566	Moderately strong
Ka3	4.82	Very important	0.405	0.566	Moderately strong
Ka4	4.82	Very important	0.405	0.566	Moderately strong
Kce1	4.55	Very important	0.522	0.690	Moderately strong
Kce2	4.55	Very important	0.522	0.690	Moderately strong
Kce3	4.55	Very important	0.522	0.690	Moderately strong
Kce4	4.64	Very important	0.505	0.646	Moderately strong
Kce5	4.55	Very important	0.522	0.690	Moderately strong
If	4.64	Very important	0.505	0.646	Moderately strong
Ef	4.82	Very important	0.405	0.566	Moderately strong
Dp1	4.73	Very important	0.467	0.606	Moderately strong
Dp2	4.82	Very important	0.405	0.566	Moderately strong
Dp3	4.73	Very important	0.467	0.606	Moderately strong
Dp4	4.73	Very important	0.467	0.606	Moderately strong
Cr1	4.82	Very important	0.405	0.566	Moderately strong
Cr2	4.55	Very important	0.522	0.690	Moderately strong
Cr3	4.64	Very important	0.505	0.646	Moderately strong

M, average expert rating; IL, Importance Level; SD, Standard Deviation; AL, Agreement Level; α_wg (1)_, Interrater agreement index.

## Discussion

In this study, we propose the Knowledge Creation for Community Resilience (KCCR) Framework. The process of identifying the factors that contribute to the KCCR Framework is strengthened by semi-structured interview and the agreement of panellists in the areas of DRR, climate change, community empowerment and development communication, on 26 factors that affect community resilience based on knowledge creation.

**FIGURE 4:**
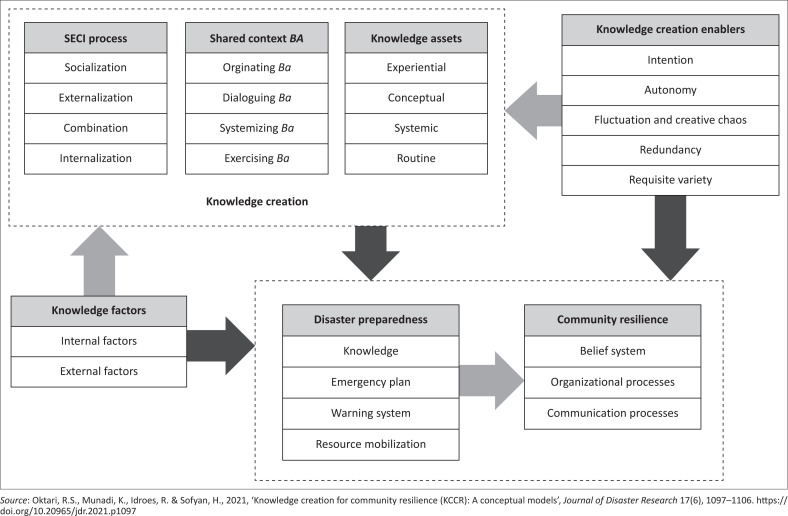
Knowledge creation for community resilience framework.

This study affirms that within the KCCR framework (see Figure 4), there are seven main elements, namely: (1) knowledge creation process/SECI process, (2) knowledge assets, (3) shared context ‘Ba’, (4) enabler of knowledge creation, (5) factors affecting knowledge, (6) disaster preparedness and (7) community resilience. These concepts were indirectly measured through several constructs, which are believed to be the expressions of the seven concepts.

The knowledge conversion process of SECI is assessed through four constructs, namely, socialisation (Kcp1), externalisation (Kcp2), combination (Kcp3) and internalisation (Kcp4). The *Ba* shared context is evaluated through four constructs, including originating Ba (Ba1), dialoguing/interacting Ba (Ba2), systemising/cyber Ba (Ba3) and exercising Ba (Ba4). Knowledge assets are appraised through four constructs, including experiential knowledge assets (Ka1), conceptual knowledge assets (Ka2), systemic knowledge assets (Ka3) and routine knowledge assets (Ka4). Enabler of knowledge creation is evaluated through five constructs, namely: intention (Kce1), autonomy (Kce2), fluctuations and creative chaos (Kce3), redundancy (Kce4) and requisite variety (Kce5). Factors affecting knowledge are assessed through two constructs, namely: internal factors (If) and external factors (Ef). Community resilience is evaluated through three constructs, namely: belief system (Cr1), organisational processes (Cr2) and communication processes (Cr3). Finally, disaster preparedness is assessed through four constructs, namely: knowledge (Dp1), emergency planning (Dp2), warning system (Dp3) and resource mobilisation (Dp4). Single head arrows or paths are used to determine causal relationship in the framework. The black arrows indicate the direct causal relationship; meanwhile, the grey arrows represent a causal relationship that indirectly supports the KCCR framework.

The development of the KCCR framework further strengthens the importance of the role of knowledge management, especially knowledge creation in the context of disaster. Several studies have demonstrated the significance of the role of knowledge, both local and scientific, to increase resilience in the face of disasters (Fujieda & Kobayashi 2013; Oktari et al. 2015, 2018). The process of how knowledge about risks is obtained and disseminated is also an introductory note for efforts to increase disaster resilience (Haruyuma & Taresawa 2014; Ikeda, Narama & Gyalson 2016; Opdyke, Javernick-Will & Koschmann 2018). Therefore, this KCCR framework is intended to provide input for the governments and practitioners in making efforts to increase community resilience. By incorporating the concept of knowledge creation in efforts to increase community resilience, it will allow individuals to make the right decisions to perform lifesaving behaviour in the face of disasters. In this context, the abilities that exist within an individual will encourage decision-making in complex situations based on their beliefs and needs (Lau & Hiemisch 2017).

To measure community resilience, it is very important to clearly define what resilience is and also to define for whom resilience is necessary (Cutter et al. 2008). In this study, the KCCR framework was developed to measure community resilience, especially at the family level towards disasters, by incorporating the theory of knowledge creation, where this concept has not been found in other previous community resilience frameworks (Sharifi 2016).

Although this study has produced a new framework, this study’s limitation is mainly concerned with the number of experts reaching consensus, which was dropping rapidly between the Delphi rounds. Experts who were not willing to take part in round II were not invited to engage in round III.

## Conclusion

This study shows that consensus is reached significantly, and panellists confirm the significance of the key constructs that are identified for increasing the resilience of knowledge creation-based societies. This study establishes a systematic, operational and multidimensional KCCR framework that combines the concepts of knowledge creation, community resilience and disaster preparedness. In practice, the KCCR framework can be used as a qualitative instrument or guidance to build community resilience based on knowledge creation. In addition, the KCCR framework can be used quantitatively as a tool for measuring community resilience in facing disasters. Using this framework, the knowledge creation process amongst family members is assessed based on the SECI process, Ba shared context, knowledge assets, knowledge creation enablers and other knowledge factors. The concept to be conveyed in this context is that the knowledge creation process that takes place amongst family members would improve the ability to share information and make decisions in order to build disaster resilience, which is assessed by the resilience factors (belief system, organisational and communication processes).

The development of the KCCR framework is the first step towards developing a more valid model. Therefore, further research is needed to evaluate the KCCR model by using appropriate analytical tools and techniques such as structural equation modelling (SEM) and analytical network processes (ANP).
